# Evaluation of Candidate Theranostics for ^227^Th/^89^Zr Paired Radioimmunotherapy of Lymphoma

**DOI:** 10.2967/jnumed.122.264979

**Published:** 2023-07

**Authors:** Diane S. Abou, Mark Longtine, Amanda Fears, Nadia Benabdallah, Ryan Unnerstall, Hannah Johnston, Kyuhwan Shim, Abbie Hasson, Hanwen Zhang, David Ulmert, Floriane Mangin, Serife Ozen, Laurent Raibaut, Stéphane Brandès, Michel Meyer, Jean-Claude Chambron, David S. Tatum, Darren Magda, Richard L. Wahl, Daniel L.J. Thorek

**Affiliations:** 1 Mallinckrodt Institute of Radiology, Washington University School of Medicine, St. Louis, Missouri;; 2 Department of Molecular and Medical Pharmacology, UCLA, Los Angeles, California;; 3 Institut de Chimie Moléculaire de l’Université de Bourgogne, UMR 6302, CNRS, Université de Bourgogne, Dijon, France;; 4 Institut de Chimie de Strasbourg, UMR 7177, CNRS, Université de Strasbourg, Strasbourg, France;; 5 Lumiphore, Inc., Berkeley, California;; 6 Department of Biomedical Engineering, Washington University in St. Louis, St. Louis, Missouri; and; 7 Siteman Cancer Center, Oncologic Imaging Program, Alvin J. Siteman Cancer Center, Washington University School of Medicine, St. Louis, Missouri

**Keywords:** radioimmunotherapy, ^227^Th, chelator, ^89^Zr

## Abstract

^227^Th is a promising radioisotope for targeted α-particle therapy. It produces 5 α-particles through its decay, with the clinically approved ^223^Ra as its first daughter. There is an ample supply of ^227^Th, allowing for clinical use; however, the chemical challenges of chelating this large tetravalent *f*-block cation are considerable. Using the CD20-targeting antibody ofatumumab, we evaluated chelation of ^227^Th^4+^ for α-particle–emitting and radiotheranostic applications. **Methods:** We compared 4 bifunctional chelators for thorium radiopharmaceutical preparation: S-2-(4-Isothiocyanatobenzyl)-1,4,7,10-tetraazacyclododecane tetraacetic acid (*p*-SCN-Bn-DOTA), 2-(4-isothicyanatobenzyl)-1,2,7,10,13-hexaazacyclooctadecane-1,4,7,10,13,16-hexaacetic acid (*p*-SCN-Bn-HEHA), *p*-isothiacyanatophenyl-1-hydroxy-2-oxopiperidine-desferrioxamine (DFOcyclo*-*p-*Phe-NCS), and macrocyclic 1,2-HOPO *N*-hydroxysuccinimide (L804-NHS). Immunoconstructs were evaluated for yield, purity, and stability in vitro and in vivo. Tumor targeting of the lead ^227^Th-labeled compound in vivo was performed in CD20-expressing models and compared with a companion ^89^Zr-labeled PET agent. **Results:** ^227^Th-labeled ofatumumab-chelator constructs were synthesized to a radiochemical purity of more than 95%, excepting HEHA. ^227^Th-HEHA-ofatumumab showed moderate in vitro stability. ^227^Th-DFOcyclo*-ofatumumab presented excellent ^227^Th labeling efficiency; however, high liver and spleen uptake was revealed in vivo, indicative of aggregation. ^227^Th-DOTA-ofatumumab labeled poorly, yielding no more than 5%, with low specific activity (0.08 GBq/g) and modest long-term in vitro stability (<80%). ^227^Th-L804-ofatumumab coordinated ^227^Th rapidly and efficiently at high yields, purity, and specific activity (8 GBq/g) and demonstrated extended stability. In vivo tumor targeting confirmed the utility of this chelator, and the diagnostic analog, ^89^Zr-L804-ofatumumab, showed organ distribution matching that of ^227^Th to delineate SU-DHL-6 tumors. **Conclusion:** Commercially available and novel chelators for ^227^Th showed a range of performances. The L804 chelator can be used with potent radiotheranostic capabilities for ^89^Zr/^227^Th quantitative imaging and α-particle therapy.

## Visual Abstract

**Figure:**
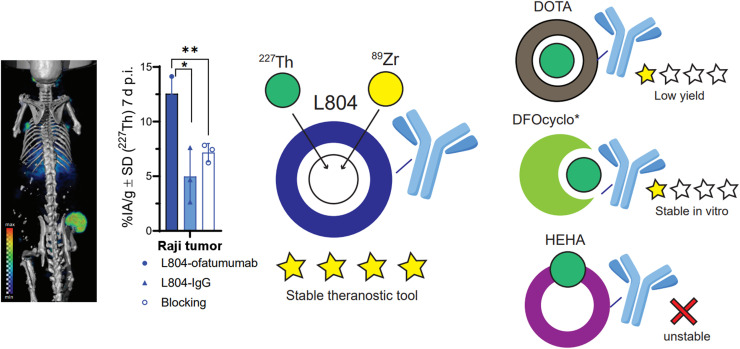

Radiotheranostic agents provide a unique ability to detect, characterize, treat, and monitor sites of disease with exceptional specificity. A persistent challenge for clinical theranostics is the development of suitably matched therapeutic and diagnostic agents that provide correlating pharmacokinetic data to guide therapeutic application. Ideally, this goal is realized in the form of a targeted agent that can be labeled with radionuclides for either imaging or therapy without other chemical changes. Radiometals must be stably bound to a molecularly specific vector (a small molecule, peptide, or antibody) to achieve localized uptake. The extended biologic residency time and longer radiologic half-life (t½) of isotopes used for antibody-based agents add a requirement for greater stability. To date, a limited number of chelates have been clinically applied, notably from the DOTA and diethylenetriaminepentaacetic acid classes (1). Advancements in radioisotopes available for theranostic applications necessitate radiologic and chemical efforts to achieve stable, safe, and effective radiopharmaceutical preparation.

Interest in treatments using α-particle–emitting isotopes with high linear-energy transfer continues to grow. A promising isotope that has been widely used to date is ^225^Ac, yet the supply of isotopically pure ^225^Ac is limited (2). Theranostic pairs for ^225^Ac radioimmunotherapy typically use ^111^In (3*,*^4^). Although these isotopes have reasonably close half-lives (2.8 and 9.8 d t½ for ^111^In and ^225^Ac, respectively), SPECT imaging presents challenges in image quantification for pharmacokinetics and dosimetry (5). Alternatively, the radiotheranostics of ^225^Ac using ^89^Zr (t½, 3.3 d) for PET have highly similar pharmacokinetics; however, different chelators are required for coordination of each isotope (6*,*^7^).

^227^Th (t½, 18.7 d) is produced from ^227^Ac (t½, 21.7 y) with a branching ratio of 98.6% and produces 5 α-particles, including from its first daughter, ^223^Ra (t½, 11.4 d) (Supplemental Fig. 1; supplemental materials are available at http://jnm.snmjournals.org). Tetravalent thorium bears a 5*f* ^0^ electronic configuration and is typically 8-, 10-, or even 12-fold coordinated. Chelation with ^227^Th has been limited to a few bifunctional ligands, such as macrocyclic DOTA (displaying inefficient labeling yields), and newer hydroxypyridinone or picolinic acid constructs. It can be challenging for such ligands to also stably complex PET isotopes (8–11), as chelation chemistries are often class-specific (transition metals, lanthanides, actinides, or other heavy metals). Cross-class metal radiolabeling involves different chemistries and mechanisms (12*,*^13^), and evaluation of single-agent theranostic precursors is ongoing (9).

In this work, we evaluated 4 antibody-chelator conjugates for in vitro and in vivo stability using ofatumumab, a human anti-CD20 antibody (14*,*^15^). The most stable ^227^Th chelator conjugate, L804, was evaluated in vivo for tumor-targeting capability in Raji tumor–bearing mice. An ^89^Zr-L804- theranostic analog was compared, as well as conventional ^89^Zr-chelating DFO. Data demonstrate long-term stability and a pharmacokinetic match between ^89^Zr tracer and ^227^Th radiotherapy, with translational potential for quantitative imaging and treatment.

## MATERIALS AND METHODS

Chemicals were from Sigma-Aldrich unless otherwise noted. Ofatumumab (Novartis) was obtained from the Washington University in St. Louis clinical pharmacy. The bifunctional chelators S-2-(4-Isothiocyanatobenzyl)-1,4,7,10-tetraazacyclododecane tetraacetic acid (*p*-SCN-Bn-DOTA) and 2-(4-isothicyanatobenzyl)-1,2,7,10,13-hexaazacyclooctadecane-1,4,7,10,13,16-hexaacetic acid (*p*-SCN-Bn-HEHA) were purchased from Macrocyclics. The desferrioxamine derivative *p*-isothiacyanatophenyl-1-hydroxy-2-oxopiperidine-desferrioxamine (DFOcyclo*-*p*-Phe-NCS) was synthesized as outlined in the supplemental materials, and macrocyclic 1,2-HOPO *N*-hydroxysuccinimide (L804-NHS) was provided by Lumiphore, Inc. Solutions were prepared with Chelex (Bio-Rad)-treated ultrapure water. ^227^Th was supplied as dried nitrates by the U.S. Department of Energy.

### Chelators and Conjugations

DFOcyclo*-*p*-Phe-NCS was prepared in 5 steps from known precursors. The synthesis and characterization are reported in Supplemental Figures 2–5 (16–20). Chelator-to-antibody ratios of 8:1 (*p*-Bn-SCN-HEHA), 5:1 (DFOcyclo*-*p*-Phe-NCS), and 4.2:1 (L804-NHS) were reacted in 0.1 M Na_2_CO_3_ (pH 9, 37°C for 1 h). L804-ofatumumab was prepared in 0.5 M NH_4_OAc, pH 5.5, with 1 mM CaCl_2_. Before ^227^Th radiolabeling, buffer was exchanged by spin desalting columns (Zeba, 40K, 0.5 mL; Thermo Scientific) to 4-(2-hydroxyethyl)-1-piperazineethanesulfonic acid (HEPES) (1 M, pH 7). Conjugate ratios were measured by capillary mass spectrometry with an Exactive Plus (Thermo Fisher). Samples were run at a resolving power of 8,750 or 17,500 at 300 m/k and analyzed by Protein Metric Intact.

### ^227^Th and ^89^Zr Radiolabeling and Purification

DOTA-ofatumumab was radiolabeled following a 2-step procedure (21*,*^22^) in which 0.925–1.85 MBq of ^227^Th(IV) nitrate dissolved in 0.2 M HCl was added to *p*-Bn-SCN-DOTA (10 mg/mL; 20 μL in 0.1 M NH_4_OAc at pH 6). We verified a pH of 6 and incubated (65°C, 1 h) under gentle shaking. After radiocomplexation, the material was conjugated to the antibody (1 mg) at pH 9 in 0.1 M Na_2_CO_3_ (37°C for 2 h); 10 μL of diethylenetriaminepentaacetic acid (50 mM) was added, then the mixture was purified twice in saline using a preconditioned spin desalting column (7 kDa).

Single-step labeling (5–7 mg/mL; 1 mg) using ^227^Th at 0.925–1.85 MBq in 0.1 M NH_4_OAc at pH 6 (HEHA) or 1 M HEPES at pH 7 (DFOcyclo* and L804) occurred under gentle shaking (37°C, 2 h) and was quenched with 10 μL of diethylenetriaminepentaacetic acid (50 mM) before purification, as above. Free ^223^Ra was adsorbed on the column resin, providing high ^227^Th isotopic purity. Radical scavengers were either ascorbic acid (10 μL, 150 mg/mL, for DOTA) or gentisic acid (0.1 M, 20 μL, for others). ^89^Zr radiolabeling of DFO-ofatumumab was conducted as previously described (15). ^89^Zr radiolabeling of L804-ofatumumab (7 mg/mL) was performed in 0.5 M NH_4_OAc with 1 mM CaCl_2_ at pH 5.5 and 37°C for 2 h, with purification as above.

### Radiopharmaceutical Quality Control

Protein concentration was determined by bicinchoninic acid assay, with more than 90% recoveries. Radiochemical yields were calculated as the ratio of initial activity to measured activity obtained after purification, using γ-counting and calibrated high-purity germanium (GEM-50195-S; Ametek) detection (for ^227^Th).

Radiochemical purity (RCP) evaluation used thin-layer chromatography (AR-2000; Bioscan) and fast protein liquid chromatography (AKTA; GE Healthcare) for both ^227^Th and ^89^Zr (Supplemental Figs. 6 and 7). Labeled antibodies were migrated on silica-coated paper with an aqueous solution of diethylenetriaminepentaacetic acid (10 mM, pH 5). A control strip of unchelated ^227^Th dissolved in NH_4_OAc displayed complete migration to the front of the strip. After thin-layer chromatography reading, samples were bisected for quantitative radioisotopic determination by high-purity germanium. Radioisotopic purity was verified after purification (Supplemental Fig. 8). Stability and purity were determined using fast protein liquid chromatography with ultraviolet light (280 nm) coupled with in-line radiodetection (Lablogic) for ^89^Zr, with 1-mL fraction collection for ^227^Th/^223^Ra γ-counting.

### In Vitro Stability Assay

^227^Th-ofatumumab constructs (100 μg/0.74–0.925 MBq) were incubated in human plasma diluted 1:10 at 37°C, under gentle shaking over 1 half-life of ^227^Th. To monitor ^227^Th dissociation from antibody, samples were surveyed by thin-layer chromatography and size-exclusion chromatography fast protein liquid chromatography every other day. Thin-layer chromatography sections and fast protein liquid chromatography fractions were γ-counted (protocol below). ^227^Th activities integrated at the antibody retention time (12–14 min) over the sum of eluted activity defines the RCP percentage of ^277^Th-ofatumumab.

Immunoreactivity was evaluated by the assay of Lindmo et al. (23). Cells were incubated with the labeled samples (∼5 ng of ^227^Th conjugate, 16.7 ± 1.33 kBq) and blocked with unlabeled ofatumumab. Raji cells (12 × 10^6^) were incubated for 1 h in phosphate-buffered saline and 1% bovine serum albumin and washed, in triplicate.

### Administration and In Vivo Distribution

The studies were approved by the Institutional Animal Care and Use Committee. For organ distribution, female 6- to 8-wk-old Swiss Webster mice (Charles River) were intravenously administered constructs through the retroorbital sinus. The animals received 5.55–9.25 kBq of ^227^Th-labeled antibody or 740 kBq of ^89^Zr analogs. Injections were adjusted with unlabeled precursor to 20 μg of antibody per injection (supplemental materials).

At the times indicated, mice were killed by CO_2_ asphyxiation and organs were γ-counted (Wizard^2^; Perkin Elmer). ^227^Th and ^223^Ra (at equilibrium) activities were determined by decomposing the γ-spectra, and percentage injected activity (%IA) per gram of tissue for ^227^Th was computed. Absolute activity per organ (Bq/g) was defined using a γ-counting methodology, applying serial dilutions of a calibrated ^223^Ra source and Bateman equation–corrected ^227^Th decay spectra (24).

### PET and PET/CT

PET imaging of SU-DHL-6–bearing mice was performed using ^89^Zr-labeled L804-ofatumumab (6.66 MBq) at 1, 2, 3, and 7 d after injection (R4 microPET; Siemens). A blocking cohort with 200 μg of unlabeled ofatumumab (2 h before tracer) was included. The scanner was calibrated with a mouse-sized cylinder phantom of aqueous ^18^F with a known activity concentration (25), energy windows of 350–650 keV, and coincidence timing of 6 ns. Corrected scanner data were reconstructed by an iterative 3-dimensional maximum a priori algorithm. Volumes of interest were defined, and %IA/mL was computed (ASIPro; Siemens).

PET/CT of the SU-DHL-6 tumor–bearing animals was performed on the Nanoscan (Mediso) on day 7. A CT acquisition of 720°, 70 kV/980 μA of 90 ms, and 4× binning was reconstructed by filtered backprojection to produce isotropic 124-μm voxels (122 × 122 × 97 mm). PET data (400–600 keV, 5-ns timing) were reconstructed using the iterative, 3-dimensional TeraTomo algorithm (4 iterations and 6 subsets; Mediso Medical Imaging Systems). Decay, attenuation, and scatter corrections were applied to quantify injected activity.

## RESULTS

**FIGURE 1.**
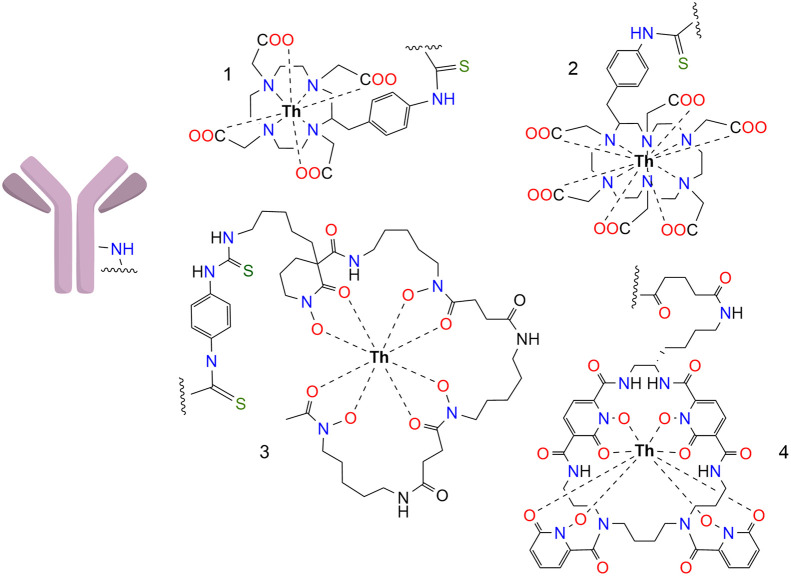
Bifunctional chelators for ^227^Th radiolabeling: ^227^Th-DOTA- (1), ^227^Th-HEHA- (2), ^227^Th-DFOcyclo*- (3), and ^227^Th-L804-ofatumumab (4). *p*-SCN-Bn-DOTA was radiolabeled using a 2-step procedure; all others were directly labeled once conjugated to antibody.

Ofatumumab, a second-generation humanized anti-CD20 antibody targeting non-Hodgkin lymphoma, was modified with 1 of 4 chelators, radiolabeled with ^227^Th, and tested for yield, purity, and stability. The bifunctional chelators considered for this study were 4-arm DOTA, HEHA, DFOcyclo*, and the L804 (Fig. 1).

### Radiochemical Yields, Purity, and Specific Activity

**FIGURE 2.**
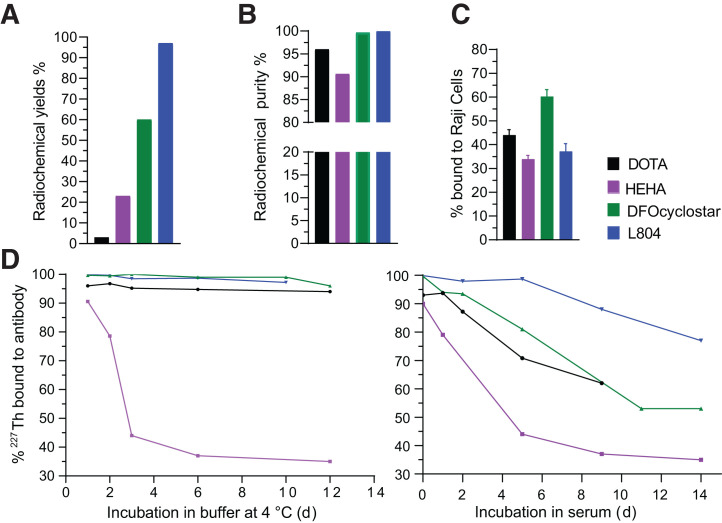
Radiopharmaceutical quality control for ^227^Th-labeled ofatumumab. All calculations were based on ^227^Th only. (A) Radiochemical yields of DOTA, HEHA, DFOcyclo*, and L804 conjugated to ofatumumab; highest yield (>95%) was obtained for L804 after purification. (B) Achieved RCPs of >95% for all except HEHA. (C) Raji cell binding showing that all ^227^Th-labeled constructs preserved moderate affinities. (D) In vitro stability of ^227^Th counts associated with antibody at 4°C in buffer (left) or human serum protein challenge at 37°C (right), over 14 d.

^227^Th labeling of DOTA-ofatumumab required a 2-step procedure first chelating ^227^Th to *p*-SCN-Bn-DOTA and then following with antibody conjugation. The final radiochemical yield reached no more than 3% because of poor conjugation efficiency. Other conjugates underwent a 1-step radiolabeling procedure resulting in radiochemical yields of 23%, 60%, and 97%, for HEHA, DFOcyclo*, and L804, respectively (Fig. 2A). The RCP of the final products was lowest for HEHA-ofatumumab (<90%), whereas the DOTA, DFOcyclo*, and L804 constructs all achieved an RCP of more than 99% (Fig. 2B; Supplemental Figs. 6 and 7). Radioisotopic purity was more than 99% for all radiopharmaceuticals, demonstrating high selectivity for ^227^Th over ^223^Ra and other daughters (Supplemental Fig. 8). The specific activities of ^227^Th-ofatumumab varied widely: 0.08 GBq/g for DOTA, 1.5–3 GBq/g for DFOcyclo*, and 8 GBq/g for L804 with 3.2 chelators per antibody (Supplemental Fig. 9). L804-ofatumumab was successfully labeled with ^89^Zr, with RCP of more than 99% (Supplemental Fig. 7), using a specific activity ranging from 330–370 GBq/g for the PET imaging study to 70–75 GBq/g for the organ distribution.

### In Vitro Stability and Immunoreactivity

Radioconjugate stability varied when challenged with human serum. HEHA demonstrated the lowest coordination capability with ^227^Th, whereas DOTA, DFOcyclo*, and L804 demonstrated stable chelation over 2 wk in buffer or plasma. DOTA and DFOcyclo* maintained adequate binding of ^227^Th ranging between 50% and 70% over 10 d of challenge (Fig. 2D; Supplemental Fig. 6). L804 presented the highest stability, with more than 80% of ^227^Th bound to the antibody after 2 wk (Supplemental Fig. 7). Binding to CD20-expressing cells indicated similar immunoreactivity for all 4 conjugates: DOTA construct binding at 44% ± 2.3% (similar to what was previously reported with rituximab (26)), HEHA at 34% ± 1.6%, DFOcyclo* at 60.2% ± 2.9%, and L804 at 32% ± 3% (Fig. 2C).

### In Vivo Distribution

**FIGURE 3.**
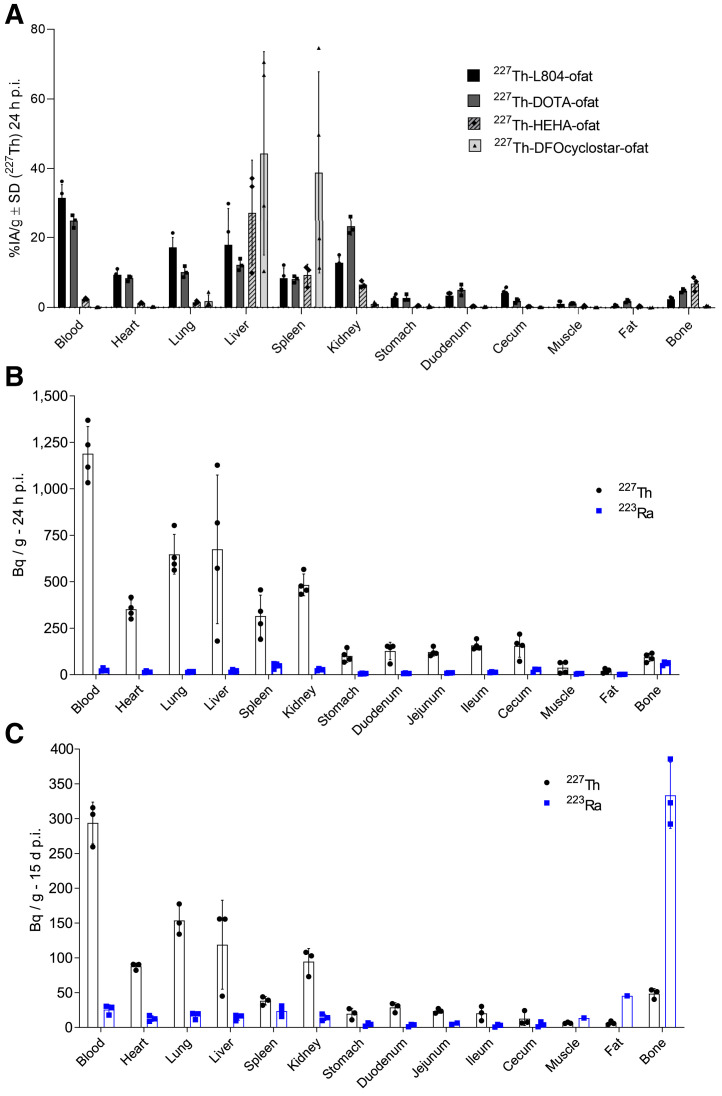
Comparative ^227^Th organ uptake in naïve mice. (A) Organ distribution of ^227^Th-DOTA-, HEHA-, DFOcyclo*-, and L804-ofatumumab 24 h after injection. Extended blood circulation was seen for DOTA and L804, compared with elevated liver and spleen uptake observed for DFOcyclo* and high liver uptake for HEHA constructs. (B and C) ^227^Th-L804-ofatumumab distribution at 24 h (B) and 15 d (C) after injection for ^227^Th and ^223^Ra. ofat = ofatumumab; p.i. = postinjection.

Acute ^227^Th stability was assessed in vivo with naïve mice at 24 h after injection (Fig. 3). ^227^Th-labeled HEHA and DFOcyclo* constructs were insufficiently stable, with elevated liver uptake (both) and spleen uptake (DFOcyclo*) (>20 %IA/g). In contrast, ^227^Th-DOTA- and ^227^Th-L804-ofatumumab presented nearly identical distributions and no significant differences in organ uptake.

The concatenated decay of ^227^Th (Supplemental Fig. 1) presents opportunities and challenges for drug development. We decomposed ^227^Th activities from daughter ^223^Ra (at equilibrium) and analyzed ^227^Th-L804-ofatumumab distribution. ^223^Ra does not decay in place, where ^227^Th accumulates, but rather recirculates and is sequestered in the skeleton (Figs. 3B and 3C) in agreement with previous reports (22*,*^27^). Initial ^227^Th uptake in lungs, liver, and kidney (>10 %IA/g) decreased over 2 wk to no more than 5%IA/g, suggesting clearance and elimination of antibody (Supplemental Fig. 10). Dosimetric evaluation of a 150 kBq/kg treatment was computed to predict human organ-absorbed doses using IDAC-Dose, version 2.1 (Supplemental Table 1) (28–30). The highest values for bone, kidney, liver, and spleen ranged from 71 to 65 mGy/MBq; heart wall and bone marrow were both 50 mGy/MBq.

### Tumor-Targeting Evaluation

**FIGURE 4.**
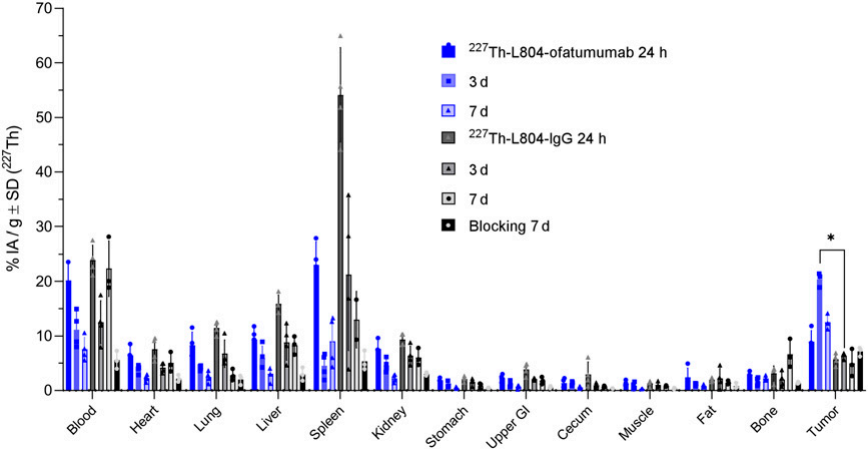
Organ distribution of ^227^Th-L804-ofatumamab and ^227^Th-L804-IgG and blocking with ofatumumab using Raji tumor–bearing mice (R2G2, female), reporting ^227^Th only (%IA/g) at 24 h, 3 d, and 7 d after injection. GI = gastrointestinal tract.

The ^227^Th-L804 conjugate was selected as the lead agent for further evaluation. We first investigated the tumor-targeting ability of ^227^Th-L804-ofatumumab in CD20–positive Raji tumors. Mice were randomized to receive ^227^Th-L804-ofatumumab, control ^227^Th-L804-IgG, or ^227^Th-L804-ofatumumab preceded by unmodified ofatumumab. The early blood signal for the unblocked group (21.6 ± 1.9 %IA/g) decreased with time to 7.5 ± 1.8 %IA/g at 7 d (Fig. 4). Control IgG uptake was significantly greater in the spleen over the course of the experiment, whereas ^227^Th tumor uptake was significantly higher for the targeted construct at all time points (to control IgG, *P* < 0.01) and to blocked group at 7 d (*P* < 0.05). Peak tumor uptake at day 3 for ^227^Th-L804-ofatumumab achieved 20 ± 1 %IA/g (Fig. 4).

### ^227^Th and ^89^Zr Theranostics

**FIGURE 5.**
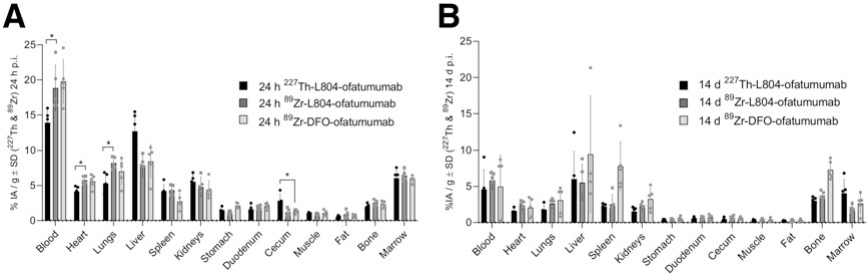
Pharmacokinetic comparison of ^227^Th-L804-, ^89^Zr-L804-, and ^89^Zr-DFO-ofatumumab in naïve female mice at 1 d (A) and 14 d (B) after injection. Significant differences were observed for blood, heart, and lung accumulation of ^227^Th-L804- vs. ^89^Zr-L804-ofatumumab at 1 d (*P* < 0.05). At 14 d, no differences were seen between ^227^Th-L804- and ^89^Zr-L804-ofatumumab. ^89^Zr bone uptake was greater for DFO (7.3 ± 1.1 %IA/g) than for L804 (^89^Zr, 3.7 ± 0.5 %IA/g; ^227^Th, 3.0 ± 0.4 %IA/g [*P* < 0.001]). p.i. = postinjection.

Having validated the stability and biologic activity of ^227^Th-L804-ofatumumab, we next tested the analogous ^89^Zr PET agent. We compared the pharmacokinetics of ^227^Th- and ^89^Zr-L804-ofatumumab and conventional ^89^Zr-DFO-ofatumumab (Fig. 5). Small but statistically significant differences were observed between ^227^Th- and ^89^Zr-L804-ofatumumab for blood, heart, lung, and cecal tissues at 1 d (Fig. 5A). At 14 d, no differences were detected (Fig. 5B). ^89^Zr-L804- and ^89^Zr-DFO-ofatumumab have highly similar distributions except for longer-term skeletal uptake; bone signal was significantly higher for ^89^Zr-DFO-ofatumumab (7.3 ± 1.3 %IA/g) than for ^89^Zr-L804-ofatumumab (3.7 ± 0.5 %IA/g). ^227^Th-L804 bone uptake was correspondingly low (3.0 ± 0.4 %IA/g). Free radiometal may also explain the increased liver and spleen values for ^89^Zr-DFO- over ^227^Th/^89^Zr-L804-ofatumumab (31). Together, these data demonstrate that use of different chelators (DFO and L804) alters radiopharmaceutical distribution to a greater degree than does exchange of radiometals.

**FIGURE 6.**
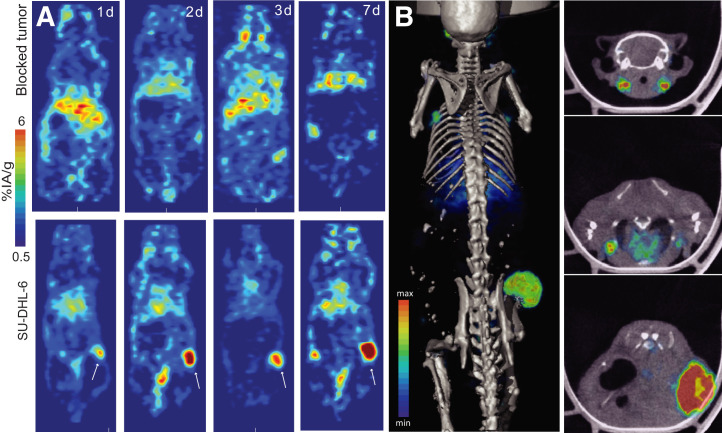
(A) Representative PET images of SU-DHL-6 tumor–bearing animal with ^89^Zr-L804-ofatumumab, with or without blocking. (B) PET/CT at 7 d, without blocking. On right are cross-sectional images of cervical lymph nodes, brachial lymph nodes, and tumor, from top to bottom.

The theranostic capability of L804-ofatumumab for ^89^Zr PET was tested in CD20–positive SU-DHL-6 xenografts (Fig. 6). Recapitulating Raji accumulation of ^227^Th-L804-ofatumumab, we observed high-contrast delineation with ^89^Zr-L804-ofatumumab and a low skeletal signal. To confirm specificity, blocking antibody was administered to a representative animal; the result was decreased tumor uptake (Supplemental Fig. 11). Metastatic invasion of lymph nodes was also visualized, along with primary tumor, and was confirmed by histologic analysis (Supplemental Fig. 12) (32).

## DISCUSSION

Radiopharmaceutical therapy is an emerging cancer treatment class. Tempering enthusiasm are concerns of off-target tissue effects and the limited availability of several radionuclides. α-particle–emitting therapy confronts both concerns because demand for a key radionuclide, ^225^Ac, greatly exceeds supply (33). Alternatives include ^223^Ra (however, receptor-specific targeting is challenging) (34) and ^227^Th (for which there is an ample stock for early-phase trials) (35*,*^36^). Labeling with ^227^Th has been achieved in the past using DOTA, octapa, and octadentate 3,2-hydroxypyridinonate structures (37), with the last of these used in clinical trials (NCT03507452 and NCT02581878). However, ^227^Th lacks suitable in vivo stability with ^89^Zr compatible chelators, potentially precluding theranostic use (22*,*^36^). Here, we chose 4 chelators from different classes to test ^227^Th coordination efficiency, stability, purity, and cancer cell receptor targeting, and we evaluated the lead conjugate as a companion ^89^Zr diagnostic.

Chelator selection was based on chemical attributes and prior experience. DOTA is a versatile chelator and has previously been used for ^227^Th coordination (22*,*^38^), which requires a 2-step procedure (26*,*^39^). The result was poor radiolabeling yields (<5%) and low specific activity (0.8 GBq/g). Previously reported ^227^Th-DOTA-antibody specific activities exceed the results of this work for ^227^Th-DOTA-ofatumumab, suggesting that antibody labeling can be further optimized (39). HEHA is a large cyclic chelator with 12 donor atoms, potentially amenable to Th^4+^ coordination (40). HEHA is an efficient in vitro chelator for ^225^Ac^3+^ (41) but has limited in vivo applicability (42). Conjugated to ofatumumab, HEHA complexed ^227^Th with limited efficiency and RCP, and data indicate that ^227^Th-HEHA-ofatumumab lacks in vivo stability.

DFOcyclo*- and L804-ofatumumab presented the most interesting radiolabeling efficiencies, purities, and specific activities with ^227^Th, in line with recent reports (43). DFOcyclo* is a linear chelator of 4 hydroxamate donors providing octadentate coordination. It presents features similar to those of DFO, with the addition of a fourth cyclic hydroxamic acid motif for additional stability to complexes embedding an 8-coordinated metal (44). We also investigated a macrocyclic approach using L804 articulated with four 1-hydroxypyridin-2-one chelators. Previously, L804 immune constructs have shown high affinity for ^177^Lu and ^89^Zr (45) and potential for actinides (46).

Both DFOcyclo* and L804 presented attractive in vitro stability, suggesting strong coordinating features for ^227^Th formulations. Surprisingly, despite excellent DFOcyclo* in vitro results, the elevated liver and spleen accumulations suggest instability of the conjugate or metal decomplexation. ^227^Th-L804-ofatumumab remained intact in vivo, as CD20-expressing tumor recognition was achieved for 2 tumor mouse models of lymphoma. ^227^Th-L804-ofatumumab organ distributions were similar to the DOTA conjugate in naïve mice, with extended blood content and low bone uptake. In vitro performance and in vivo utility indicate that L804 is an effective chelator of ^227^Th for radiopharmaceutical applications.

γ-spectroscopic analysis of the radiolabeled material showed selective ^227^Th labeling with insignificant ^223^Ra. However, concatenated decay leads to production of daughters over time, complicating quality control and in vivo evaluation (47). ^227^Th-L804-ofatumumab was administered with high radionuclidic purity, and in vivo ingrowth of ^223^Ra was notable for its skeletal redistribution anticipated from ^223^RaCl_2_ distribution in mice (27*,*^48^) and other ^227^Th conjugates (49). ^227^Th-L804-ofatumumab organ distribution over 2 wk indicates clearance from off-target organs including lungs, liver, spleen, and kidneys. Predicted human dosimetry showed that bone, kidney, and spleen may receive the highest absorbed doses for activity administrations of 150 kBq/kg. We computed low bone marrow dose estimates (<2 Gy). Considering stable ^227^Th coordination, the magnitude of tumor activity–delivery, and dosimetry, ^227^Th-L804 may drive further interest in radioimmunotherapy.

Finally, we addressed the theranostic potential for quantitative imaging using ^89^Zr-L804-ofatumumab. Subtle but significant differences were measured in blood (early time points) versus ^227^Th, and these differences resolved at 2 wk; otherwise, a nearly identical distribution was observed. In contrast, the increasing bone uptake with DFO conjugate indicated inadequate long-term stability. PET imaging of ^89^Zr-L804-ofatumumab further confirmed effective chelation of ^89^Zr by L804, displaying—with clear contrast—primary tumor SU-DHL-6 and diseased lymph nodes and showing low skeletal uptake.

## CONCLUSION

L804 is the most stable and versatile chelator of those tested, providing facile coordination of ^227^Th and ^89^Zr. Stable chelation of ^227^Th was demonstrated and applied for tumor-targeted delivery across 2 lymphoma models. These data support the further development of ^227^Th/^89^Zr antibody theranostics using this chemically identical precursor.

## DISCLOSURE

Financial support was received from NIH NCI (R01CA229893, R01CA240711, and R01EB02925901 to Daniel Thorek), P30 CA091842, the Children’s Discovery Institute of Washington University in St. Louis and St. Louis Children’s Hospital (to Diane Abou), the Centre National de la Recherche Scientifique (CNRS), the Conseil Régional de Bourgogne through the Plan d’Action Regional pour l’Innovation (program PARI II “Pharmaco-imagerie et agents théragnostiques”), the European Regional Development Fund (FEDER), and the University of Strasbourg. Floriane Mangin acknowledges the Université de Bourgogne for a postdoctoral fellowship. Darren Magda and David Tatum own intellectual property relating to L804 and are employees of Lumiphore, Inc. (Berkeley, CA). No other potential conflict of interest relevant to this article was reported.

## Notes

KEY POINTS **QUESTION:** Can we stably achieve antibody radiolabeling using an identical precursor for both ^89^Zr imaging and ^227^Th therapy? **PERTINENT FINDINGS:** L804 is the most stable and versatile chelator of 4 tested for coordination of ^227^Th and ^89^Zr as a theranostic pair. **IMPLICATIONS FOR PATIENT CARE:** These data support the development of suitably matched therapeutic and diagnostic agents that provide correlating pharmacokinetic and pharmacodynamic data to guide therapeutic application.
